# Special Issue: Alcohol Policy and Public Health—Contributing to the Global Debate on Accelerating Action on Alcohol

**DOI:** 10.3390/ijerph17113816

**Published:** 2020-05-28

**Authors:** Charles D. H. Parry, Niamh Fitzgerald

**Affiliations:** 1 Alcohol, Tobacco and Other Drug Research Unit, South African Medical Research Council, PO Box 19070, Tygerberg 7405, South Africa; 2 Department of Psychiatry, Faculty of Medicine and Health Sciences, Stellenbosch University, PO Box 241, Cape Town 8000, South Africa; 3 Institute for Social Marketing and Health, Faculty of Health Sciences and Sport, University of Stirling, Stirling FK9 4LA, UK; niamh.fitzgerald@stir.ac.uk

In July 2018, under our guidance, *IJERPH* put out a call for papers to be considered for publication in a Special Issue on “Alcohol and Public Health”. This was motivated by the reports of mixed success regarding the achievements by member states on the objectives of the Global Strategy to Reduce the Harmful Use of Alcohol [1,2]. In the Global Strategy [1], member states are specifically encouraged to more systematically institute evidence-based strategies to address alcohol problem and to consider action in some or all of the areas below:

1. Leadership, awareness and commitment.
2. Health services’ response.
3. Community action.
4. Drink-driving policies and countermeasures.
5. Availability of alcohol.
6. Marketing of alcoholic beverages.
7. Pricing policies.
8. Reducing the negative consequences of drinking and alcohol intoxication.
9. Reducing the public health impact of illicit alcohol and informally produced alcohol.
10. Monitoring and surveillance.

Our intention as guest editors was to provide a platform for the publication of papers that would advance action around how best to progress alcohol control at local, national, and regional/global levels to reduce alcohol-related harms. We were also acutely aware of initiatives by the alcohol industry including through international trade agreements, to thwart national alcohol policy efforts.

In September 2018, WHO launched its SAFER initiative [3] further focusing national and international efforts on five high-impact strategic priorities for the prevention of alcohol-related death and disability and promotion of health and development. The five measures are: *Strengthen* restrictions on alcohol availability; *Advance* and enforce drink driving countermeasures; *Facilitate* access to screening, brief interventions, and treatment; *Enforce* bans or comprehensive restrictions on alcohol advertising, sponsorship, and promotion; and *Raise* prices on alcohol through excise taxes and pricing policies.

As a follow-up to the political declaration of the third high-level meeting of the General Assembly on the prevention and control of non-communicable diseases in May 2019, the WHO Director General was requested to report to the Seventy-third World Health Assembly in 2020, through the Executive Board, on the implementation of WHO’s Global Strategy to Reduce the Harmful Use of Alcohol during the first decade since its endorsement, and the way forward [4]. Several actions followed, including (i) the hosting of a second WHO Forum on Alcohol, Drugs and Addictive Behaviours in June 2019 in Geneva, (ii) regional consultations with member states on the implementation of the WHO Global Strategy to Reduce the Harmful Use of Alcohol since its endorsement, and the way forward, and validation of country information collected through a country-level survey on the implementation of the Global Strategy, (iii) a web-based consultation on a discussion paper that had been prepared on the implementation of the WHO Global Strategy to Reduce the Harmful Use of Alcohol since its endorsement, and the way forward, and (iv) the WHO prepared an addendum to the report referred to above summarizing inputs received since the release of the preliminary report/discussion paper.

At the 146th session of the WHO Executive Board in February 2020, consideration was given to the report and its addendum. According to newspaper reports, “A group of member states, led by Bangladesh, Indonesia, Iran, Sri Lanka and Thailand, have been pressing the WHO Executive Board (EB) to support the rapid development of a tough new WHO approach to a health risk that claims 3 million lives a year—to replace the failed strategy of a decade ago. The member states, supported by civil society groups combatting alcohol abuse and non-communicable diseases, wanted to see WHO embrace the development of binding “international instruments”, such as measures to reduce digital and cross-border marketing of alcohol products to adolescents” [5]. It was reported, however, that such measures “met with stiff opposition from a cluster of countries that have big alcohol industry lobbies, including Japan and the United States” [5]. In the end, a decision was adopted at the Executive Board in favour of identifying alcohol as a new public health problem and requesting the Director General to: (i) develop an action plan (2022–2030) to effectively implement the WHO Global Alcohol Strategy as a public health priority; (ii) develop a technical report on cross-border alcohol marketing, advertising and promotional activities, including those targeting youth and adolescents; (iii) resource the work on alcohol harm and policy solutions adequately; and (iv) review the WHO Global Alcohol Strategy and report to the 166th session of the Executive Board in 2030 for further action [6]. This decision was the result of a compromise pushed through by several countries with powerful industry interests that wanted the removal of the request to launch a working group exploring the development of an international instrument for alcohol control [6]. Given the underlying sources of this pushback, it is likely that the industry will persuade some governments to seek further compromises when this is debated by member states at the World Health Assembly in May 2020.

Over the roughly 18 months that the call for articles was open with *IJERPH*, 19 articles were submitted, 15 (79%) of which were finally accepted for publication. This Special Issue, completed in early May 2020, follows the 146th session of the WHO Executive Board but precedes the virtual 73rd World Health Assembly in May 2020, at which the attending member states will be invited to consider the same or an updated report. It also comes at a time at which there is an increasing demand for good information to inform the implementation of effective alcohol control measures at a country level, especially from Low and Middle Income Countries (LMICS) [7]. Table 1 shows a breakdown of how the 15 papers line up with the 10 areas for action outlined in the 2010 WHO Global Strategy to Reduce the Harmful Use of Alcohol [1], the six areas of the SAFER initiative [3], the various levels where action may be taken, the country where the study was undertaken, whether the paper includes a focus on industry behaviour, the location/country of the research, the type of study and the main findings. Six of the articles were of a qualitative nature. 

Considering the focus of the articles in comparison with WHO guidance, it is notable that none of the articles touched significantly on the areas of leadership, awareness and commitment; health services’ response; reducing the public health impact of illicit and informally produced alcohol; or monitoring and surveillance in general. Five of the fifteen articles included a focus on industry behaviour, in four instances the alcohol industry, and in one instance the advertising industry, but with a focus on alcohol advertising. In all cases, the industry behaviours reported were not supportive of public health or efforts to effectively address alcohol-related harms. The articles clearly illustrate that addressing alcohol-related harms involves multi-level governance including organizational, local, state/provincial, and national levels. Despite rising levels of consumption in LMICs, most of the papers (12/15) focused on high income countries, with just one each from Lebanon [13] and South Africa [20]. The most common methodology used in the studies published in this Special Issue was qualitative case studies, followed by pre-post designs (Table 1).

Six of the studies [8,15,17,20,21,22] evaluated the effectiveness of an intervention. Of these, two [8,20] found showed a meaningful effect, with only one [8], looking at strategies to reduce the availability and promotion of alcohol on or near university campuses, finding a statistically significant effect. Two studies of the night-time environment in Australia did not find significant effects. One found that monthly police-recorded serious assaults did not significantly change within safe night precincts or local government areas following the introduction of restrictions on sale of higher strength alcohol after midnight [15]. The second found that having premises pay tailored license fees based on an assessment of the risks arising from each premises was not associated with a reduction on the incidence of assaults linked to drinking in licensed premises [17]. It is striking that neither intervention reduced the temporal availability of alcohol, whereas several recent studies have shown that closing premises earlier, especially after midnight, is strongly associated with reductions in assaults [23,24,25]. The results of the two studies here, raise questions about the likely effectiveness of efforts to shape, rather than reduce, the supply of alcohol in the night-time environment.

The other studies were more descriptive and did not test any interventions. Nonetheless, they provide some useful conclusions, among others, that:

- “state innovativeness in traffic safety policies, organizational size, and professionalism of state highway department increased the likelihood of a state adopting a more comprehensive bundle of DUI laws” [11],
- “accountability was an important factor for understanding why there is a tension between the intentions of licensing legislation and the way it is enacted in practice” [12],
- “minimum risk for cardiovascular disease was achieved at or below alcohol use levels of 10g/day ethanol” [18], and
- “individual-level knowledge of the alcohol-cancer link was associated with higher levels of support for pricing policies, specifically, setting a minimum unit price per standard drink of alcohol” [19].

The five papers which included a focus on alcohol and advertising industry practices [9,10,13,14,16] add to a growing evidence base about such practices. For example, Knai et al. [9] found that both the production and uptake of responsibility pledges by responsibility deal partners in the UK, including organisations with commercial interests, were largely driven by the interests of partners themselves, and this made it possible for them to resist change. A somewhat similar finding emerged in a study conducted in Lebanon [13], where it was noted that industry representatives argued against evidence-based policies in reducing alcohol harm experienced by youth. A study by Hessari and colleagues [10] found that alcohol industry funded organisations were significantly more likely to tweet about behavioural aspects of drinking and less likely to mention (breast) cancer risk [10]. A second study by Hessari et al. involved a review of 39 case studies published by the advertising industry evaluating the effect of alcohol advertising campaigns [14]. Campaigns were found to increase consumption outcomes, and to often target younger drinkers, women, new/lapsed drinkers and heavy drinkers. This study provides data from the industry’s own documents, that contradicts key claims about the purpose and aim of alcohol advertising. Finally, Paixão and Mialon [16] found that the alcohol industry in Portugal works in partnership with health authorities belonging to the national task force responsible for planning alcohol control policies and it uses its influence to emphasize the role alcohol plays in Portuguese culture as a way to disregard evidence on control policies from other countries. Together, the studies highlight the importance of limiting the involvement of the alcohol industry and its affiliates, with their substantial conflicts of interest, in alcohol policy forums.

The final paper in the issue [22] outlines a set of studies, covering an impressive range of research questions and scope, to explore the potential consequences of the introduction of minimum unit pricing in Scotland. Whilst Scotland is not the first nation to introduce minimum pricing (or even minimum unit pricing), the evaluation may be among the most comprehensive ever of a single public policy. The findings of the studies using this methodology will have important implications not just for the decision on whether or not to continue with minimum unit pricing in Scotland, but in contributing to global understanding. As such, it will have implications for other nations implementing or considering similar controls.

## Figures and Tables

**Table 1:** Description of the areas covered by the research in the 15 articles in the special issue, the country of the study, the level of the intervention, the type of study and main findings.

Authors	GS Areas *	SAFER Areas **	Industry Behaviour	Level of Intervention or Study Focus	Country	Type of Study	Main Findings
Kypri et al. [8]	5, 6	S, E	-	Local	New Zealand	Pre-post design	Strategies to reduce the availability and promotion of alcohol on or near university campuses can reduce the incidence of health and social harms
Knai et al [9].	-	-	Alcohol industry	Organisational	UK	Systems analysis	The production and uptake of pledges by Responsibility Deal partners were largely driven by the partners themselves, enabling these wider systems to resist change
Hessari et al. [10]	6, 7	E, R	Alcohol industry	Organisational	UK	Qualitative case study	Alcohol industry bodies were less likely to tweet about alcohol marketing, advertising and sponsorship; alcohol pricing; and physical health harms
Hwang and Berry [11]	3	A	-	State, provincial	USA	Panel analysis	State innovativeness in traffic safety policies, organizational size, and professionalism of state highway department increases the likelihood that a state will adopt a more comprehensive bundle of DUI laws
Wright et al. [12]	5	S	-	State, provincial	UK	Qualitative case study	Accountability is an important factor for understanding why there is a tension between the intentions of licensing legislation and the way it is enacted in practice
Nakkash et al. [13]	3, 4, 5, 6	S, A, E, R	Alcohol industry	National	Lebanon	Qualitative study	Three themes emerged: Inadequacy of current alcohol control policies; weak governance and disregard for rule of law as a determinant of the status quo; and diverting of responsibility towards “other” stakeholders. Industry representatives argued against evidence-based policies using time-worn strategies identified globally
Hessari et al. [14]	6	E	Advertising industry	Organisational	Global	Analysis of case studies	Most found that alcohol advertising campaigns increased consumption-related outcomes. Some campaigns targeted women, heavy drinkers and often targeted younger drinkers. The data present evidence of a causal relationship between advertising and consumption.
Taylor et al. [15]	5	S	-	State, provincial	Australia	Time series analysis	Monthly police-recorded serious assaults did not significantly change within safe night precincts or local government areas following the introduction of liquor restrictions.
Paixão and Mialon [16]	-	-	Alcohol industry	Organisational, national	Portugal	Qualitative analysis of case studies	The industry works in partnership with health authorities, belonging to the national task force responsible for planning alcohol control policies. Alcohol plays a role in Portuguese culture as a way to disregard evidence on control policies from other countries
Nepal et al. [17]	5	S	-	State, provincial	Australia	Pre- post analysis	Found a small decrease in all assaults, but no change in the incidence of assault attributed to drinking in licenses premises following implementation of risk-based licensing
Sherk et al. [18]	7	--	-	National	Australia, Canada	Burden of disease analysis	Minimum risk for cardiovascular disease was achieved at or below alcohol use levels of 10g/day ethanol. Consumption levels resulting in “no added” risk from drinking were found to be between 10 and 15 g/day, by country, gender, and scenario.
Weerasinghe et al. [19]	5, 7	S, R.	-	Local	Canada	Pre-post analysis	Support for pricing and availability policies was low overall; however, increases in individual-level knowledge of the alcohol-cancer link was associated with higher levels of support for pricing policies, specifically, setting a minimum unit price per standard drink of alcohol
Bowers et al. [20]	5	S	-	State, provincial	South Africa	Case study analysis	Although not statistically significant, the number of alcohol outlets and the density per 1000 population declined by about 12% and 34% between 2008 and 2016. Illegal outlets were still more likely to be located in more deprived areas.
Gilmore et al. [21]	7	R	-	National	Australia	Interrupted time series analysis	None of the gender and age-specific population-based rates indicated a significant immediate or lagged association with the tax in terms of reducing the national chlamydia rate among young people. However, found an immediate decrease in test positivity rates for 25–34-year-old males that remained detectable up to a lag of six months and a decrease at a lag of six months for 15–24-year-old males following the tax.
Beeston et al. [22]	7	R	-	National (Scotland)	UK	Mixed methods	A method is presented and data will only become available at a later stage.

* (1) Leadership, awareness and commitment, (2) Health services’ response, (3) Community action, (4) Drink-driving policies and countermeasures, (5) Availability of alcohol, (6) Marketing of alcoholic beverages, (7) Pricing policies, (8) Reducing the negative consequences of drinking and alcohol intoxication, (9) Reducing the public health impact of illicit alcohol and informally produced alcohol, (10) Monitoring and surveillance. ** Strengthen restrictions on alcohol availability, Advance and enforce drink driving counter measures, Facilitate access to screening, brief interventions and treatment, Enforce bans or comprehensive restrictions on alcohol advertising, sponsorship, and promotion, Raise prices on alcohol through excise taxes and pricing policies.
